# Quantification of gastric tube perfusion following esophagectomy using fluorescence imaging with indocyanine green

**DOI:** 10.1007/s00423-022-02546-0

**Published:** 2022-05-17

**Authors:** Philipp von Kroge, Detlef Russ, Jonas Wagner, Rainer Grotelüschen, Matthias Reeh, Jakob R. Izbicki, Oliver Mann, Sabine H. Wipper, Anna Duprée

**Affiliations:** 1 Department of General, Visceral and Thoracic Surgery, University Medical Center Hamburg-Eppendorf, Hamburg, Germany; 2 Department for the Development of Applications, Institute for Laser Technology, University Ulm, Ulm, Germany; 3 Department of Vascular Surgery, University Medical Center Innsbruck, Innsbruck, Austria

**Keywords:** Indocyanine green, Gastric tube perfusion, Anastomotic leakage, Esophagectomy, Fluorescent imaging

## Abstract

**Introduction:**

Anastomotic leakage (AL) remains a prevalent and life-threatening complication after esophagectomy. Gastric tube perfusion assessment using indocyanine green fluorescence imaging (ICG-FI) has been published in several studies and appears to be a promising tool to reduce AL rates by changing the surgical approach, namely by an intraoperative evaluation of the anastomosis localization.

**Methods:**

In this study, gastric tube perfusion was quantified by using ICG-FI in 20 high-risk patients undergoing esophagectomy. From a time-dependent fluorescence intensity curve, the following three parameters were evaluated: slope of fluorescence intensity (SFI), background subtracted peak fluorescence intensity (BSFI), and time to slope (TTS).

**Results:**

The values between pyloric region and tip showed a similar downward trend and SFI and BSFI significantly correlated with the distance to the pyloric region. SFI and BSFI were significantly decreased at the tip of the gastric tube. The placement of anastomosis in an area with homogenous fluorescence pattern was correlated with no AL in 92.9% of cases. An inhomogeneous fluorescence pattern at anastomotic site was a risk factor for the occurrence of an AL (*p* < 0.05). Reduction of perfusion up to 32% using SFI and up to 23% using BSFI was not associated with AL.

**Conclusion:**

ICG-FI can be used to quantify the gastric tube perfusion by calculating SFI, BSFI, and TTS. The anastomosis should be created in areas with homogeneous fluorescence pattern. A reduction in blood flow of up to 32% can be accepted without causing an increased rate of insufficiency.

## Introduction

Esophageal cancer is one of the most frequently observed causes of cancer-related death worldwide [1]. To date, esophagectomy remains an important part of multimodal treatment approaches for resectable esophageal cancer [4]. However, with a prevalence of 7 to 30%, anastomotic leakage (AL) is a potentially life-threatening complication after esophagectomy [5–7]. Next to a prolonged duration of hospital and intensive care unit stay, AL is also associated with an increased postoperative mortality [8, 9]. The most common risk factors for AL are tension on the anastomosis, surgical technique, location of anastomosis, surgeons experience, active smoking, corticosteroid therapy, and comorbidities like heart failure, renal insufficiency, and arterial hypertension [5, 8, 10–13]. Since another important risk factor for AL is a poor local blood supply, the gastric tube in particular is at high risk for AL due to its anatomical conditions with an arterial perfusion exclusively via the right gastroepiploic artery and the possibility of venous congestion [5]. Therefore, intraoperative evaluation of gastrointestinal perfusion remains a difficult challenge in surgery. Subjective parameters for perfusion evaluation are bleeding from resection margin, pulsation of supplying vessels, and tissue color [14]. In addition, some technologies like laser Doppler flowmetry have been investigated in the past few years, which, however, could not prevail [15].

Indocyanine green fluorescence imaging (ICG-FI) is a promising tool for intraoperative perfusion evaluation. ICG is a fluorescent dye, which was originally developed for cardiac diagnostics in 1956 [16]. The dye is hepatically eliminated, and major side effects have rarely been described [17]. Furthermore, the operation time is not extended by FI [18].

There are several studies using ICG for gastric tube evaluation, which have shown a benefit regarding the occurrence of AL [18–22]. In addition, two meta-analyses evaluating gastric tubes using ICG-FI showed an advantage in terms of reducing the incidence of AL [23, 24]. In contrast, another meta-analysis resulted in comparable AL rate with or without the use of ICG-FI for intrathoracic anastomosis in patients undergoing totally minimally invasive esophagectomy [25].

However, the use of ICG remains mostly subjective, although various parameters for quantifying ICG-FI have already been described [26, 27]. Nonetheless, there is a lack of evidence, especially for measuring gastric tube perfusion, and only a few but promising studies have been published to date [28, 29].

In addition, our recently published data regarding the validation of quantification of fluorescence imaging in animal experiments are promising. ICG-FI was able to adequately predict gastric tube microperfusion and correlated with fluorescent microspheres, which represent the gold standard in experimental microperfusion assessment [30]. The aim of our pilot study is to quantify the gastric tube perfusion using FI and to validate previously published data in high-risk patients undergoing esophagectomy.

## Methods

### Patient characteristics

In this single-center pilot study, we included 20 patients aged ≥ 18 years with resectable esophageal carcinoma undergoing conventional thoracoabdominal or cervicothoracoabdominal esophagectomy. Open surgery was chosen due to insufficient feasibility of laparoscopic surgery due to various previous abdominal surgeries. Regarding pre-existing comorbidities, there were no exclusion criteria. Furthermore, there were no exclusion criteria regarding previous operations or therapies.

For reconstruction, a gastric tube was created in all patients. The greater curvature was dissected preserving the right gastroepiploic artery and vein. The left gastric artery and the left gastroepiploic vessels were resected radicularly. Gastric tube was created with a linear stapler device using purple magazines of 60 mm length (Endo GIA™, Medtronic, Dublin, Ireland). Intrathoracic anastomoses were created using a 28-mm circular stapling device, and cervical anastomosis was performed using a double-row hand-sewn technique.

All patients were preoperatively assessed according to the physical status classification system of the American society of anaesthesiologists (ASA PS) [31].

If an AL was suspected, endoscopy, computed tomography, or both were performed.

### Fluorescence imaging and quantification

After completing gastric tube construction, we performed an intraoperative FI of the gastric tube before moving it to the thoracic cavity using 0.02 mg/kg bodyweight ICG (Verdye 5 mg/ml Diagnostic Green GmbH, Aschheim-Dornach, Germany) applied via a central vein catheter. Central vein catheter was flushed with 20 ml of saline after ICG administration. For FI, we used the “SPY Elite” fluorescence imaging system (Novodaq). The device was placed vertically over the tissue and the optimal distance was determined using the integrated laser aiming aid.

The gastric tube perfusion was intraoperatively assessed based on the fluorescence pattern. If there was a demarcation line between subjectively well-perfused areas and those areas with impaired perfusion, it was marked by suture or ink. Whenever possible, the anastomosis was placed in areas with good perfusion pattern.

**Fig. 1:**
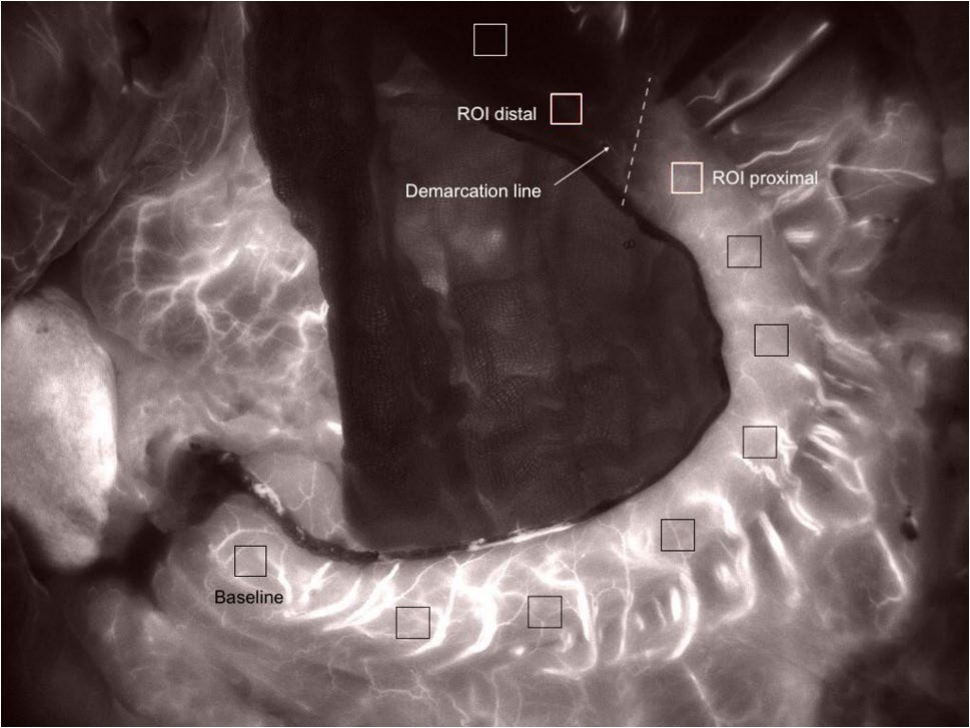
Fluorescence image after gastric tube creation. Figure 1 shows the fluorescent image of a gastric tube. There is homogenous fluorescence up to a line of demarcation (dashed line). Ten regions of interest (ROI) are marked (squares) starting at the baseline at the pyloric region. In each ROI a time-dependent fluorescence intensity curve was generated

For objective quantification, the imaging was postoperatively analyzed using the custom-made software “*Meteroarchive VCL LLS Fluoreszenzangiographie V 1.0*” (LLS GmbH) on basis of gray-scale analysis. The tube was virtually divided into up to ten regions of interest (ROI) starting at the prepyloric region and finishing at the tip depending on length of the tube. If a line of demarcation was visible, regions next to it were selected likewise (Fig. 1).

In every ROI, a time-dependent fluorescence intensity curve was generated. Based on this curve, three parameters were calculated as previously described [32, 33]:

- The slope of fluorescence intensity (SFI): This parameter represents the maximum increase of the calculated curve during the first pass of the ICG.
- The background subtracted peak fluorescence intensity (BSFI): The initial fluorescence intensity is subtracted from the maximum intensity during first passage of ICG.
- The time to slope (TTS): TTS represents the time between ICG injection and first increase of fluorescence intensity in the ROI.

**Fig. 2:**
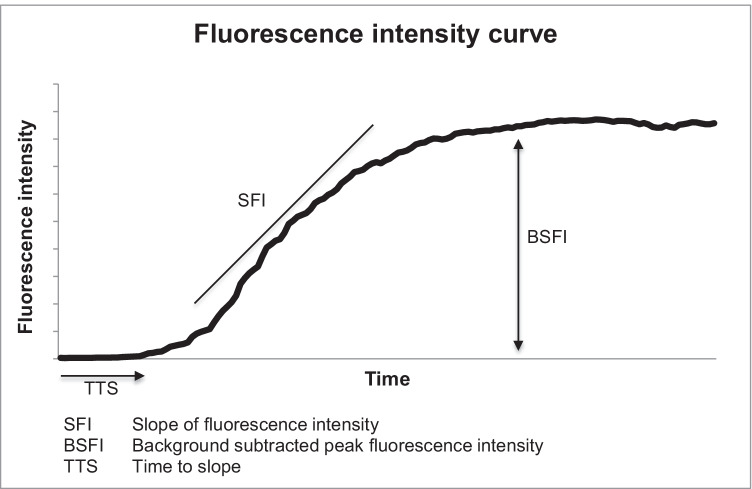
Fluorescence intensity curve with SFI and BSFI and TTS. Figure 2 shows a time-dependent fluorescence intensity curve calculated from a region of interest in a well-perfused area of the gastric tube. The three calculated parameters SFI, BSFI, and TTS are demonstrated. SFI describes the slope of the curve. For calculation of BSFI, background fluorescence intensity is subtracted from peak fluorescence intensity. TTS represents the time from ICG injection to onset of slope of fluorescence intensity

The fluorescence intensity of the prepyloric region was defined as the optimal perfusion (baseline). The time-dependent fluorescence intensity curve and schematic representation of the calculation of SFI, BSFI, and TTS are shown in Fig. 2.

### Statistics

Statistical analysis was performed using SPSS statistical software package 27.0 (IBM). A value of *p* < 0.05 was considered statistically significant. Continuous variables are presented as the means and standard deviations (SD). Variance homogeneity was tested using Levene’s test. If inhomogeneous, analysis of variance (ANOCA) with Welch’s test was carried out. Dunnetts’s T3 test was carried out for a post hoc analysis of the effect differentiation between the areas. The correlation was calculated using Spearman’s rank correlation coefficient. Cohort data were analyzed using chi^2^ test, Fischer’s exact test, and *t*-test.

## Results

### Patient characteristics and clinical outcome

A total of 20 patients were included in this pilot study. Of those, 14 patients were male and the mean patient age was 64.$\pm$ 13.1 years. Four patients were scored ASA II, 14 patients ASA III, and two patients ASA IV. Eleven patients had previously received neoadjuvant treatment. Two patients underwent prior liver transplantation and eight patients had a history of another malignancy. Major thoracic surgery was performed in three patients prior esophagectomy and major abdominal surgery in six patients (Table 1).

**Table 1:** Patients’ characteristics. *SD*, standard deviation; *ASA PS*, physical status classification system of the American society of anaesthesiologists

Characteristics	No anastomotic leakage (*n* = 13)	Anastomotic leakage (*n* = 7)	*p*-value
Age (years) [mean ± SD]	66 ± 7.1	62.0 ± 19.1	*p* = 0.635
Sex (male/female)	10/3	4/3	*p* = 0.613
Anastomosis: Thoracic/cervical	9/4	5/2	*p* = 0.664
ASA PS classification: I–II/III–IV	2/11	2/5	*p* = 0.587
Neoadjuvant treatment	7	5	*p* = 0.526
Cardiac disease	8	5	*p* = 0.642
Active smoking	9	6	*p* = 0.613
Chronic renal insufficiency	2	1	*p* = 0.730

Fourteen patients underwent thoracoabdominal esophagectomy with intrathoracic stapler anastomosis whereas a hand-sewn cervical anastomosis was performed in six patients. In 14 patients, anastomosis was placed in homogenous fluorescent areas proximal of the line of demarcation. In these patients, AL occurred in case.

**Fig. 3:**
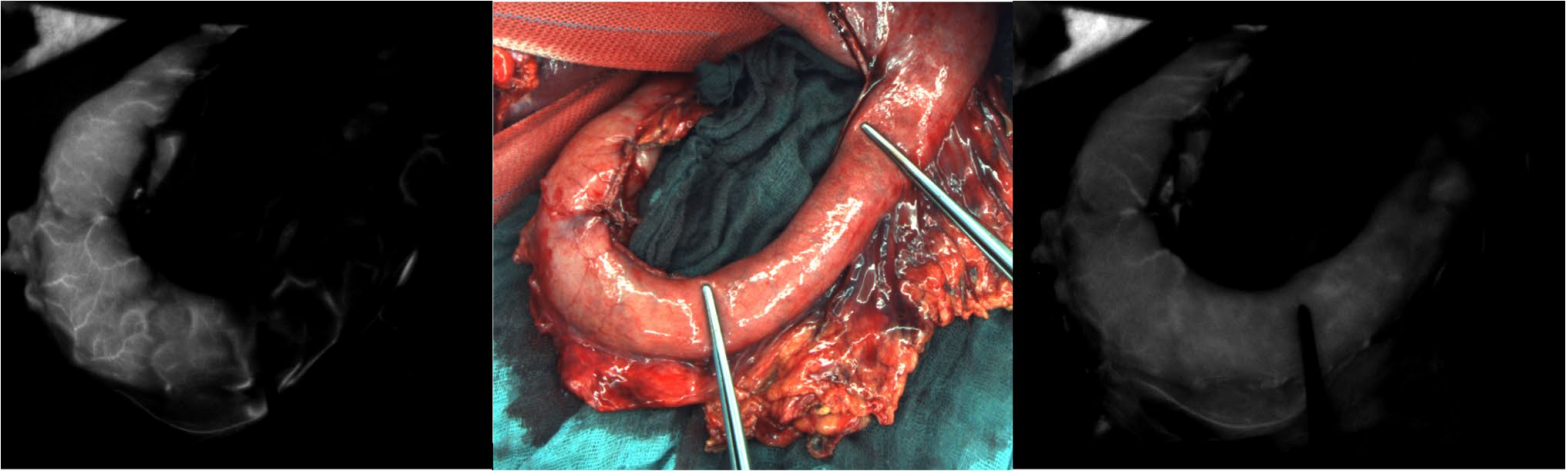
Image with gastric tube showing impaired perfusion. All the pictures show the same gastric tube. The middle shows the macroscopic picture of the gastric tube; the two lines of demarcation resulting from the FI-ICG (left and right imaging) are each marked with one forceps. Macroscopically, there are no signs of impaired perfusion while FI-ICG shows two clear demarcation lines

Overall, AL occurred in seven patients (35%). In one case, the fluorescent line of demarcation was close to the pylorus. The anastomosis had to be placed in an area with compromised perfusion. On the second postoperative day, the gastric tube showed severe ischemia in upper endoscopic evaluation, and hence, a discontinuity resection had to be performed. Due to previous bowel resection, a colonic interposition was not possible (for the macroscopic picture and ICG-FI imaging of this case, refer to Fig. 3).

Due to the length of the tube, anastomosis had to be placed in the area of the demarcation in four patients. These patients developed AL and were each treated with endoscopic vacuum sponge therapy.

Two patients with cervical anastomosis suffered from AL, which could be treated conservatively. Due to length limitation, anastomosis had to be placed at the very tip of the tube in these patients.

Patients with AL and without AL showed no significant differences regarding ASA PS, age, location of anastomosis location (thoracic vs. cervical), cardiac diseases, renal insufficiency, active smoking, and neoadjuvant treatment. Moreover, macroscopically inhomogeneous fluorescence pattern at anastomotic side was an independent risk factor for the occurrence of an AL (*p* < 0.05). The overall-30-day mortality was 0%.

### Assessment of tissue perfusion by ICG-FI

The quantification of the gastric tube perfusion was successfully assessed by using SFI, BSFI, and TTS (Fig. 2). If a subjective demarcation line between macroscopically well-perfused areas and areas of compromised perfusion was visible, quantification was performed in areas adjected to the line (Fig. 1).

#### SFI

SFI decreased with an increasing distance from the prepyloric region. SFI was significantly lower at the tip of the gastric tube. Mean SFI at the pyloric region was 85.1 $\pm$ 38.3, mean SFI at the tip was 8.3 $\pm$ 7.7, and the mean ratio between pyloric region and tip was 0.14 $\pm$ 0.19 (*p* < 0.001). Thus, the perfusion level at the tip was 14% compared to the pylorus.

There was a demarcation line in 18 cases. SFI showed a significant difference between the adjacent areas. Mean SFI pre-demarcation was 48.8 $\pm$ 34.3 and mean SFI post-demarcation was 16.2 $\pm$ 13.3 (*p* < 0.05). The perfusion post-demarcation was reduced by 66% compared to the pre-demarcation area. Post-demarcation area and tip of the gastric tube showed no significant different SFI values. SFI values of the pre-demarcation line were significantly lower compared to the values at the pyloric region.

There was a significant correlation between an increasing distance from the pyloric region and SFI values; Spearman’s correlation coefficient was 0.732 (*p* < 0.001).

#### BSFI

BSFI showed similar results. BSFI was significantly lower at the tip of the gastric tube. Mean BSFI at the pyloric region was 173.0 $\pm$ 61.4, mean BSFI at the tip was 36.1 $\pm$ 32.7, and mean ratio between pyloric region and tip was 0.23 $\pm$ 0.20 (*p* < 0.001). Perfusion level at the tip was 22% compared to the pylorus. Mean BSFI pre-demarcation was 117.4 $\pm$ 62.4 and mean BSFI post-demarcation was 47.6 $\pm$ 30.46 (*p* < 0.05). The perfusion in post-demarcation area was reduced by 58% compared to the pre-demarcation area. Post-demarcation area and the tip of the gastric tube showed no significant different BSFI values. BSFI values in the pre-demarcation region were not significantly lower compared to the values at the pyloric region.

**Fig. 4:**
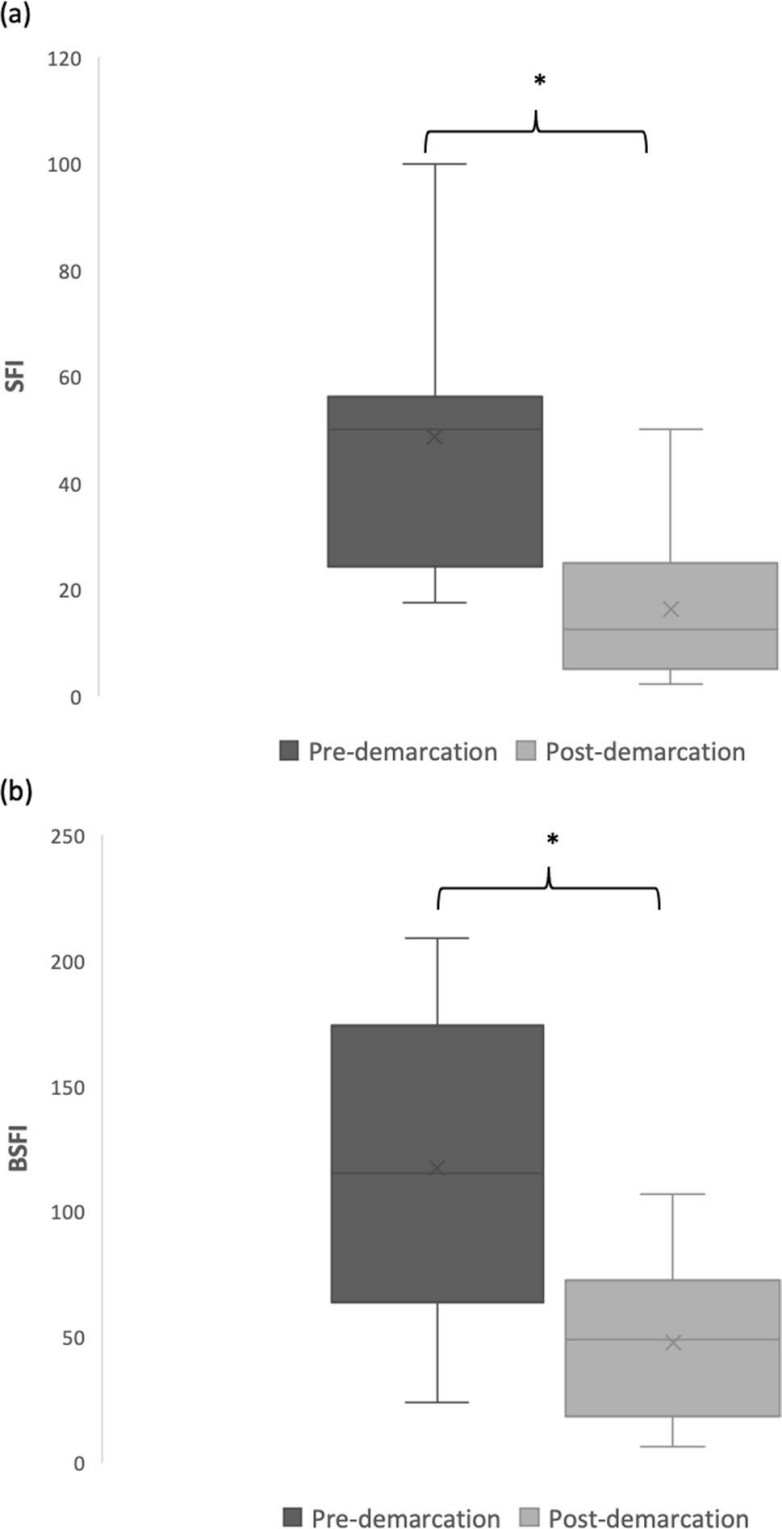
Slope of fluorescence intensity (SFI) and background subtracted peak fluorescence intensity (BSFI) around the demarcation line. **a** SFI values pre-demarcation and post-demarcation are shown as a boxplot. The difference pre-demarcation and post-demarcation reaches significance (* = *p* < 0.001). **b** BSFI values pre-demarcation and post-demarcation are shown as a boxplot. The difference pre-demarcation and post-demarcation reaches significance (* = *p* < 0.001)

**Fig. 5:**
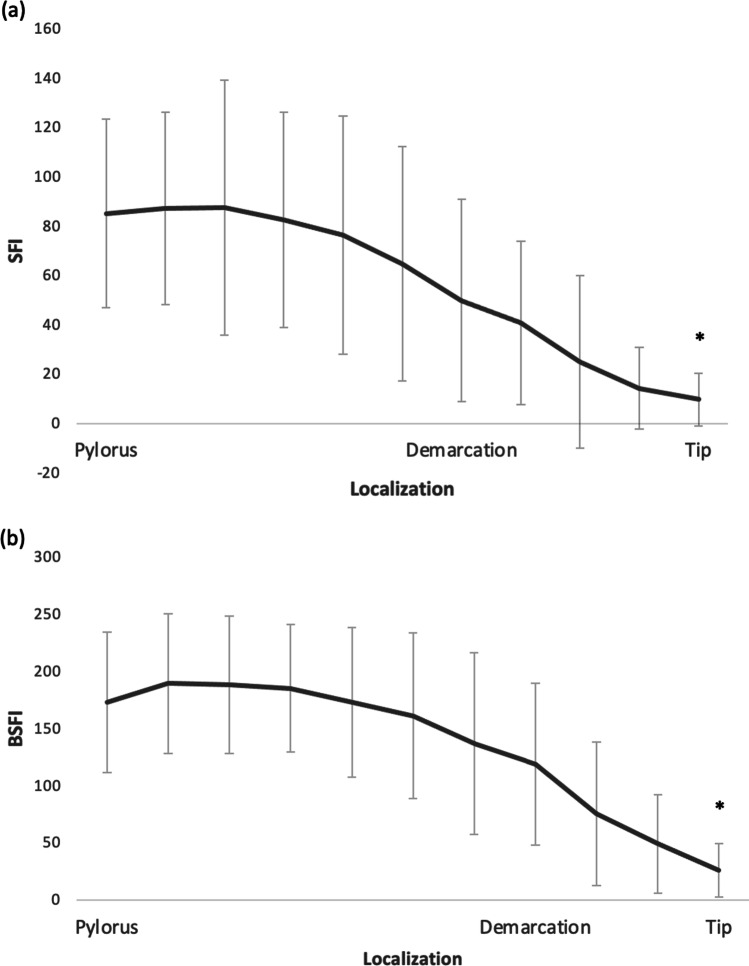
Mean slope of fluorescence intensity ratio (SFI) and mean background subtracted peak fluorescence intensity (BSFI) with standard deviation (SD). **a** SFI values with SD of the gastric tube starting at pylorus. The difference between pylorus and the tip of the tube reaches significance (* = *p* < 0.001). Demarcation line (dashed line) is drawn at mean pre-demarcation value. **b** BSFI values with SD of the gastric tube starting at pylorus. The difference between pylorus and the tip of the tube reaches significance (* = *p* < 0.001). Demarcation line (dashed line) is drawn at mean pre-demarcation value

There was a significant correlation between increasing distance from the pyloric region and BSFI values; Spearman’s correlation coefficient was 0.709 (*p* < 0.001). The differences of the values at demarcation line are shown in Fig. 4. Mean SFI and BSFI values are shown in Fig. 5.

#### TTS

TTS is represented as a ratio to the pylorus as a baseline value. Perfusion onset was significantly prolonged at the pre-demarcation area, post-demarcation area, and at the tip (1.26 $\pm$ 0.30, 1.37 $\pm$ 0.29, 1.58 $\pm$ 0.48; *p* < 0.05). There was no significant difference between pre- and post-demarcation areas. Spearman’s correlation coefficient was 0.564 (*p* < 0.001).

## Discussion

Recently, the use of intraoperative ICG-FI in esophagectomy with gastric pull-up and the possible reduction in the rate of anastomotic leaks have been increasingly described [18, 19, 22, 34]. However, the intraoperative use of ICG-FI is mostly based on subjective assessment criteria.

First, by quantifying the ICG-FI and correlating anastomotic leakage rate of 20 patients undergoing esophagectomy with gastric pull-up, we were able to show that the calculated parameters SFI, BSFI, and TTS could sufficiently predict the gastric tube perfusion. The measured SFI and BSFI values decreased significantly with increasing distance to the tip of the gastric tube, indicating impaired perfusion. In addition, a significantly impaired perfusion could be demonstrated distal of the visible demarcation line. Remarkably, in all six patients, anastomotic leakage occurred if anastomosis could not be placed in areas with homogenous fluorescent pattern. In addition to the subjective use of the ICG-FI, we were able to objectify the significant impairment of perfusion in this area through quantification.

Our findings are consistent with previously published studies that describe a demarcation line in the setting of ICG-FI during esophagectomy. Karampinis et al. studied a group of 35 patients undergoing esophagectomy. An intraoperative FI was performed and the gastric tube was divided into a well-perfused “optizone” and a poorly perfused area. In the majority of patients (33/35), anastomosis was placed in the “optizone.” Of those, AL occurred in only one patient. Thus, Karampinis and colleague reported on a significantly lower AL rate in the ICG-FI group (3% vs. 18.2%) compared to a retrospective control group [18]. In addition, Zehetner et al. observed an impressive reduction in AL rate when the anastomosis was placed in a well-perfused area identified by using ICG-FI (2% vs. 45%) [19]. Furthermore, a meta-analysis of 19 studies involving 1186 patients confirmed the decrease in AL rate by using ICG-FI. Subgroup analysis revealed an AL rate of 6.3% of anastomosis that was placed in a well-perfused area. Another meta-analysis reported similar results with a 69% reduction in AL rate [23]. Moreover, Casas et al. analyzed 32 studies with a total of 3171 patients undergoing minimally invasive thoracoabdominal esophagectomy. Of those, an ICG-FI was performed in 381 patients. In contrast to the previously published data, the authors showed equal AL rates in both groups. However, the included studies were overall heterogeneous, and only one included study reported on the impact of ICG-FI-guided surgery [25].

In addition to the detecting of a demarcation line, Nerup et al. also quantified gastric tube perfusion by using a previously published algorithm [35]. As a result, quantification of ICG (q-ICG) showed different locations for the best anastomotic position compared to white light evaluation and ICG-FI without quantification [28]. Similar to our results, the demarcation line was detected using ICG-FI. In our study, SFI showed significantly lower perfusion level pre-demarcation; thus, the different location of ICG-FI and q-ICG can be explained by the different perfusion levels in the selected areas. In addition, we correlated the perfusion pattern with the rate of AL. Reduction of SFI and BSFI was not necessarily associated with AL. Besides, we identified the lack of homogenous fluorescence pattern as an independent risk factor for AL, which is consistent with previously reported data [22].

TTS was significantly longer with increasing distance to the pylorus indicating a reduced perfusion level. This is in line with the 90-s rule proposed by Kumagai et al. A delayed homogenous fluorescence pattern after 90 s was associated with higher AL rate [34]. Moreover, a delayed fluorescent enhancement at the tip after injection (> 98 s) was associated with anastomotic leakage [36]. In addition, TTS significantly correlated with increasing distance to the pylorus.

Despite the use of ICG-FI occurrence, AL remains high in the present study. The main cause is certainly the high-risk patient population with above-average illnesses, mainly caused by the selection criteria for a primary open surgical approach since 16 of 20 patients were preoperatively classified as ASA III or IV.

The quantification of ICG-FI using SFI and BSFI has previously been described for myocardial perfusion measurement. The results were correlated with fluorescent microspheres as gold standard of experimental quantification of tissue microperfusion [32]. In our previous animal studies on the quantification of ICG-FI using SFI, BSFI, and TTS with simultaneous correlation with fluorescent microspheres, the calculated parameters could sufficiently predict gastric tube perfusion [30]. In the current study, we were able to confirm and translate our animal experimental results in the human gastric tube. Both values continuously decreased with increasing distance to the pyloric region. Ishige et al. describe a similar trend in the perfusion measurement of the gastric tube using FImax for quantification of ICG-FI in 20 patients, with no AL occurring in the study population [29]. In fact, the FImax describes a similar parameter as the BSFI. In contrast to Ishige et al., we calculated a cut-off value regarding the AL rate. Interestingly, a perfusion reduction of up to 32% in SFI and 23% in BSFI was not associated with AL. Thus, our quantification tool can also represent a relevant decision-making innovation in cases that do not have a visible demarcation line.

This study has some limitations mainly due to the limited cohort size and a missing control group. Furthermore, the majority of patients included in this study had pre-existing comorbidities which consecutively were associated with high risk for perioperative morbidities and for AL in particular. Therefore, our study cohort is only partially representative for assessment of AL.

In conclusion, the calculated parameters SFI, BSFI, and TTS can predict the local tissue perfusion of the gastric tube. Since the demarcation line indicates a significant impairment of perfusion even after quantification, the anastomosis should definitely be created in areas with homogeneous fluorescence pattern. A reduction in blood flow of up to 32% can be accepted without causing an increased rate of AL. These preliminary results have to be confirmed in future prospective and randomized trials.
